# Resting-state perfusion in motor and fronto-limbic areas is linked to diminished expression of emotion and speech in schizophrenia

**DOI:** 10.1038/s41537-023-00384-7

**Published:** 2023-08-12

**Authors:** Nicole Gangl, Frauke Conring, Andrea Federspiel, Roland Wiest, Sebastian Walther, Katharina Stegmayer

**Affiliations:** 1 Translational Research Center, University Hospital of Psychiatry and Psychotherapy, Bern, Switzerland; 2 Graduate School for Health Sciences, University of Bern, Bern, Switzerland; 3 Support Center of Advanced Neuroimaging (SCAN), University Institute of Diagnostic and Interventional Neuroradiology, Inselspital, Bern, Switzerland

**Keywords:** Limbic system, Schizophrenia, Schizophrenia

## Abstract

Negative symptoms (NS) are a core component of schizophrenia affecting community functioning and quality of life. We tested neural correlates of NS considering NS factors and consensus subdomains. We assessed NS using the Clinical Assessment Interview for Negative Symptoms and the Scale for Assessment of Negative Symptoms. Arterial spin labeling was applied to measure resting-state cerebral blood flow (rCBF) in 47 schizophrenia patients and 44 healthy controls. Multiple regression analyses calculated the relationship between rCBF and NS severity. We found an association between diminished expression (DE) and brain perfusion within the cerebellar anterior lobe and vermis, and the pre-, and supplementary motor area. Blunted affect was linked to fusiform gyrus and alogia to fronto-striatal rCBF. In contrast, motivation and pleasure was not associated with rCBF. These results highlight the key role of motor areas for DE. Considering NS factors and consensus subdomains may help identifying specific pathophysiological pathways of NS.

## Introduction

Negative symptoms (NS) form a core component of schizophrenia, characterized by deficits in emotion and behavior, hampering treatment compliance and leading to social withdrawal, reduced quality of life and poor functional outcome^1,2^.

Previous studies investigated NS as a heterogeneous single dimension, often disregarding the hypothesized specific mechanistic differences of NS subdomains including blunted affect, alogia, anhedonia, asociality and avolition^3^. In fact, investigations of the structure of NS led to a comprehensive agreement to classify NS as two distinct factors: diminished expression (DE) and motivation and pleasure (MAP)^4–7^. DE includes the NIMH-MATRICS consensus subdomains blunted affect and alogia and is characterized by reduced outward expression of emotion and speech. The second factor MAP, includes anhedonia, asociality and avolition and represents symptoms related to impairments in motivation, goal-directed behavior and decision-making^6^. Different terms have been used in the literature for both NS factors DE (e.g. expressive factor, Expressive Deficit domain) and MAP (e.g. apathy, avolition-apathy, experiential factor)^8^. Throughout the manuscript, we will be referring to the two factors as diminished expression (DE), and motivation and pleasure (MAP). Importantly recent studies report partly distinct cognitive, behavioral and neural correlates for each factor. For example, deficits in DE are mostly linked to cognitive impairments and everyday life skills^8–10^. Conversely, MAP is strongly linked to poor interpersonal relationships, functional outcome and depression^4,7,10,11^.

To date, neural correlates of NS in schizophrenia are not fully understood. Previous studies investigated NS as a heterogeneous single dimension, rarely were neural correlates of DE explored as a NS factor. Hitherto evidence resulting from both functional and structural imaging (rs-fMRI, sMRI) suggests, that blunted affect is associated with aberrant limbic and paralimbic brain activity which includes the amygdala, parahippocampal, anterior cingulate, orbitofrontal, medial and ventrolateral prefrontal, premotor, motor and parietal areas, as well as, the fusiform gyrus (FFG)^12–16^. Alogia, the second subdomain of DE has been linked to abnormal activity within the anterior cingulate cortex (ACC)^17^ and areas of the basal ganglia, including pallidum and caudate^18,19^. In contrast, more studies focused on the neural correlates of MAP by applying rs-fMRI, sMRI and investigating the anticipation of reward in different fMRI tasks. Several studies and meta-analytic evidence suggest hypoactivation of the ventral striatum as a neuronal correlate of MAP (i.e. during the anticipation of reward)^20–25^. Further evidence suggests the dorsal caudate as an additional striatal area to be involved in reward anticipation^21,26^. Besides the central role of striatal areas, some studies also linked MAP severity to reduced activation in the cingulate cortex and insula^23^, as well as, in the ventromedial prefrontal cortex^22^ and the inferior frontal gyrus^27^. Importantly, studies on NS subdomains do not directly compare these subdomains in respect of the neuronal correlates. Thus, studies focusing on both NS factors to examine neuronal correlates are needed.

In sum, areas relevant for cognitive and emotional processing in motor and fronto-limbic networks seem to play an essential role in the pathophysiology of DE. In contrast, MAP is thought to be associated with changes in prefrontal-striatal areas^28^. The majority of these studies however present differences in the blood-oxygen-level-dependent (BOLD) signal between rewarding and non-rewarding stimuli. Studies reflecting absolute activations in different brain areas and NS subdomains are rare. In particular, arterial spin labeling uses arterial water as a tracer to measure resting-state cerebral blood flow (rCBF). Therefore, ASL results in an absolute quantification of rCBF with high spatial and temporal resolution rendering rCBF an essential correlate of brain functioning^29^. Thus while BOLD contrast primarily detects changes that indirectly reflect changes in CBF, ASL perfusion techniques directly quantify CBF, and are believed to be directly linked to neuronal activity in contrast to BOLD signal changes^30^. Therefore, ASL measures may exhibit decreased inter-subject variability compared to BOLD. Few studies have used rCBF quantifications to investigate NS as a uniform construct with conflicting results. In detail, one study found a positive association between NS and increased perfusion in the left inferior temporal gyrus and insula^31^, while two other studies detected decreased perfusion in bilateral superior temporal gyrus (STG) and left middle and inferior frontal gyrus (IFG)^32,33^. The first two studies used the Positive and Negative Syndrome Scale (PANSS) to assess NS, while Pinkham and colleagues used the global score of the Scale for the Assessment of Negative Symptoms (SANS). The current conceptualization of NS however suggests the use of newer assessments such as the Clinical Assessment Interview for Negative Symptoms (CAINS) and the Brief Negative Symptom Scale (BNSS) to measure NS, or omitting the global scales when using the SANS^34–36^. Further, more recent evidence advocates the examination of the five consensus subdomains separately, which might fit the psychometric data more adequately^34–36^. Regarding NS and brain perfusion, only two small studies focused on NS factors. In particular, Liemburg and colleagues reported less task-related rCBF in fronto-parietal, motor and thalamic regions^37^ associated with MAP. Schneider and colleagues showed higher striatal perfusion associated with MAP performing region of interest analyses^38^. To our knowledge, neither study has investigated neural correlates of the NS factors nor the five consensus subdomains using ASL whole brain resting-state perfusion analyses.

The current study therefore aims to investigate the association of severity of DE, MAP and the related five consensus subdomains and whole brain changes in rCBF in schizophrenia patients. We conceptualize NS considering the most recent guidelines^34–36^ using the CAINS and SANS. Based on the current literature, we hypothesize, that DE and its related subdomains will be associated with altered perfusion in motor and fronto-limbic areas, while MAP and its related subdomains will be associated with aberrant perfusion in prefrontal-striatal networks.

## Results

Patients showed moderately severe psychotic symptoms according to PANSS total scores. Patients and controls did not differ in age, sex, or duration of education. Within 24 h of the testing eight patients were treated with antidepressants, eight were taking benzodiazepines and 15 received anticonvulsant medication.

### Negative symptom factor DE is associated with perfusion in the supplementary motor area and the anterior cerebellum

**Fig. 1:**
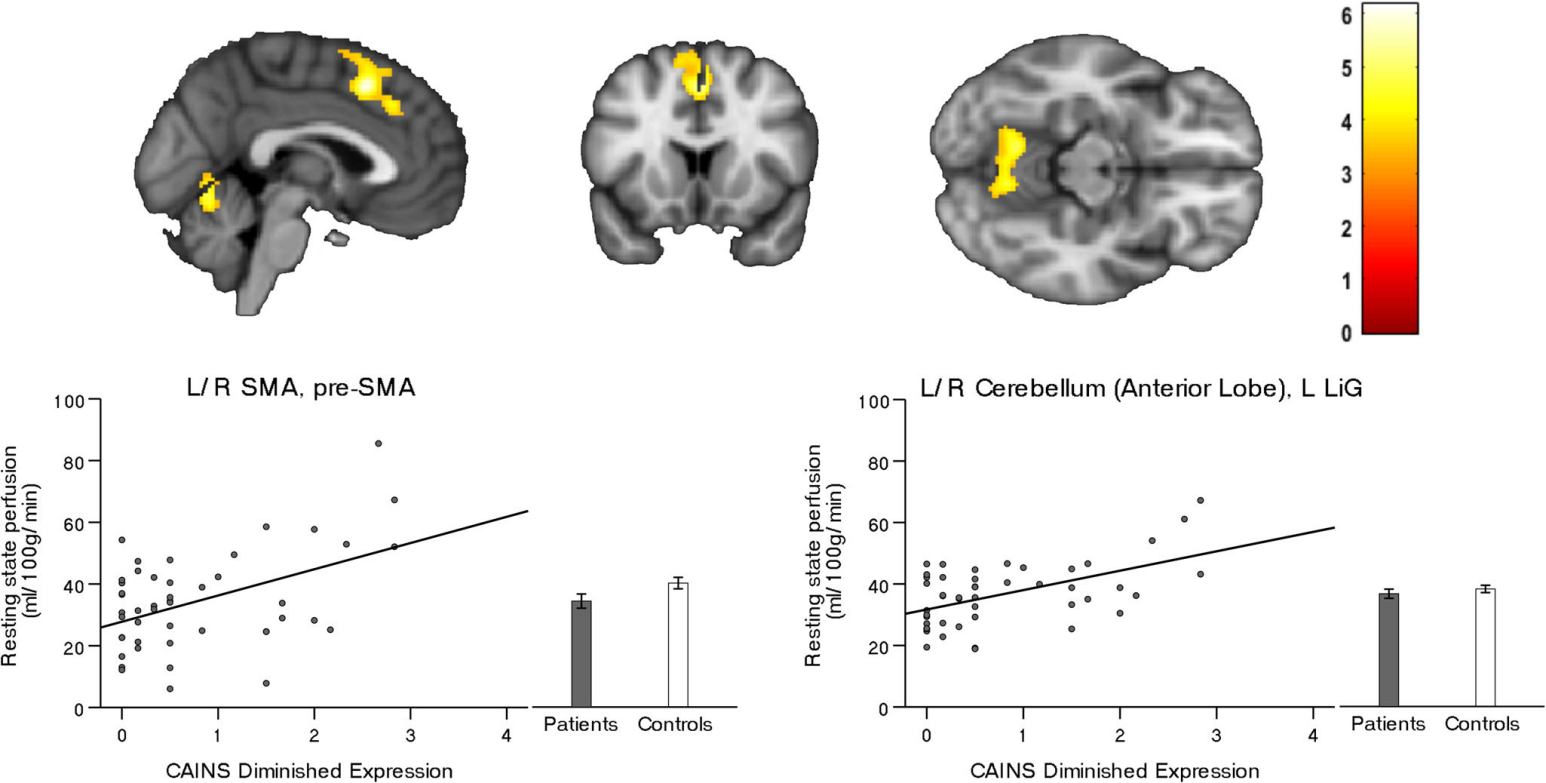
Resting-state perfusion associated with Diminished Expression. Correlations between resting-state perfusion and negative symptom severity according to the Clinical Assessment Interview for Negative Symptoms (CAINS). Depicted clusters are significant at *p*_(FWE-corr)_ < 0.05 at peak or cluster level. SMA Supplementary motor area, LiG Lingual gyrus.

We observed an association between severity of DE and rCBF bilaterally in the pre-supplementary and supplementary motor area (SMA) as well as the anterior lobe of the cerebellum and the cerebellar vermis (Fig. 1). Specifically, severe reduced emotional and speech expression (DE) was associated with higher perfusion in these regions. Conversely, we observed no significant association between brain perfusion and the second main negative symptom factor (MAP). In addition, comparing patients and controls independent of NS identified lower rCBF in patients within the right planum temporale (see Table S2).

### Negative symptom subdomain alogia is associated with perfusion within the supplementary motor area, ventral premotor area and the anterior cerebellum

**Fig. 2:**
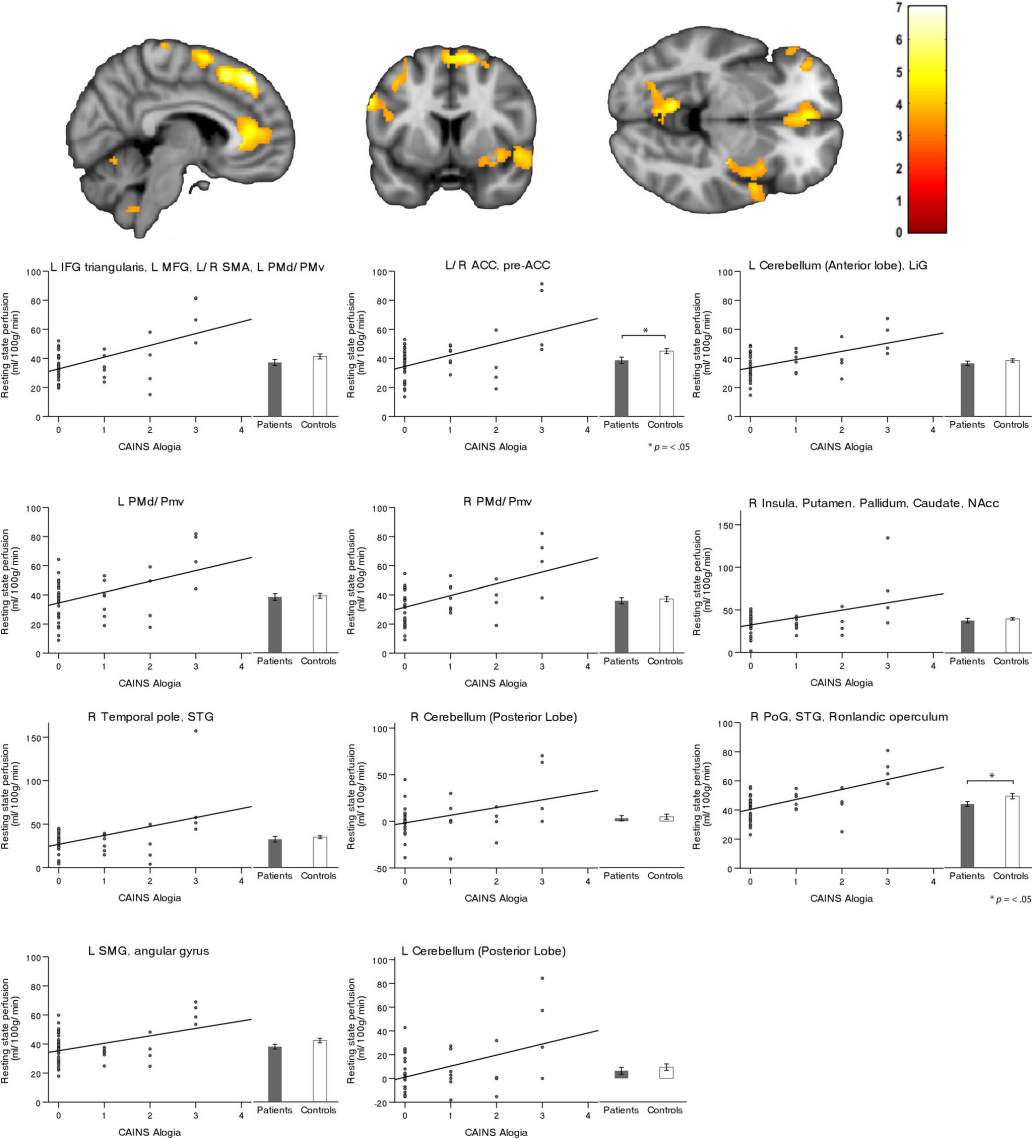
Resting-state perfusion associated with Alogia. Correlations between resting-state perfusion and negative symptom severity according to the Clinical Assessment Interview for Negative Symptoms (CAINS). Depicted clusters are significant at *p*_(FWE-corr)_ < 0.05 at peak or cluster level. IFG Inferior frontal gyrus, MFG Middle frontal gyrus, SMA Supplementary motor area, PMd Dorsal premotor area, PMv Ventral premotor area, ACC Anterior cingulate cortex, LiG Lingual gyrus, NAcc Nucleus accumbens, STG Superior temporal gyrus, PoG Postcentral gyrus, SMG Supramarginal gyrus.

**Fig. 3:**
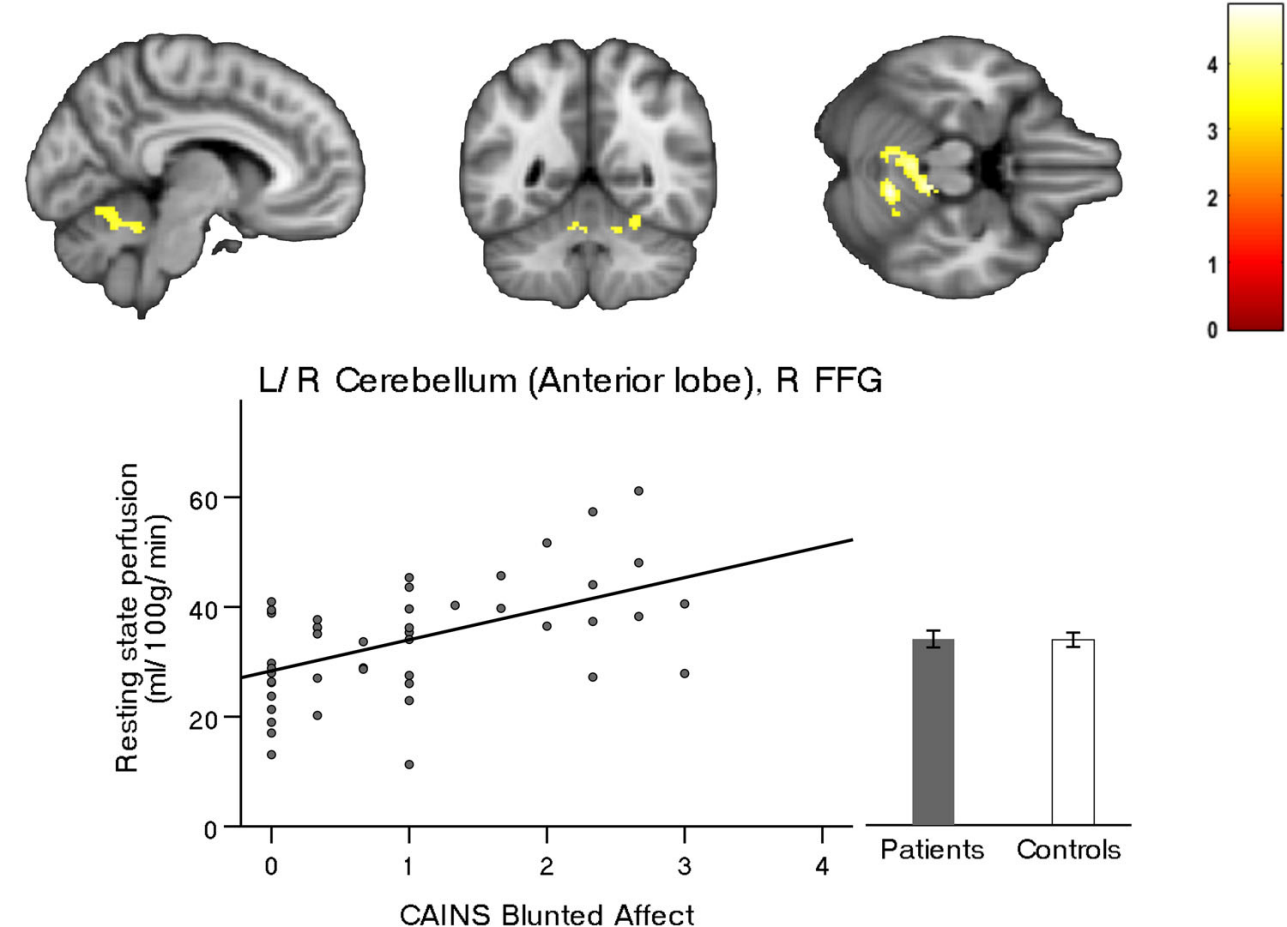
Resting-state perfusion associated with Blunted affect. Correlation between resting-state perfusion and negative symptom severity according to the Clinical Assessment Interview for Negative Symtoms (CAINS). Depicted clusters are significant at *p*_(FWE-corr)_ < 0.05 at peak or cluster level. FFG Fusiform gyrus.

Similarly, and in accordance with the main negative symptom factor DE we detected an association between severity of the subdomain alogia and rCBF bilaterally most prominent within the anterior and posterior lobe as well as the cerebellar vermis and again the pre-SMA/SMA cluster. In addition, we found an association within the bilateral anterior cingulate cortex (ACC), the right insula, head of the caudate, putamen and nucleus accumbens, as well as within the triangularis part of the left inferior frontal gyrus (IFG), dorsal and ventral premotor area (PMd/PMv), postcentral gyrus (PoG) and lingual gyrus, left supramarginal gyrus (SMG), rolandic operculum and superior temporal gyrus (STG) (Fig. 2, Table 1:2). Importantly, these results differ only marginally when applying either scale (SANS or CAINS, see Table S1). In addition, the subdomain blunted affect was associated with brain perfusion bilaterally within the anterior lobe and vermis of the cerebellum and the right FFG (Fig. 3, Table 1:3). However, we detected no associations when testing the three MAP subdomains (anhedonia, asociality, and avolition).

**Table 1:** Perfusion in brain regions associated with dimensions and consensus domains of negative symptoms according to CAINS.

Brain region		*P* (FWE-corr)					
	*k*_E_	Cluster	Peak	*T*	MNI coordinates (*x*, *y*, *z*)		
**1. Association with severity of CAINS Diminished Expression**							
L/R SMA, pre-SMA	892	0.001	0.009	6.16	2	18	48
			0.208	4.84	8	40	48
			0.222	4.81	6	−2	68
L/R Cerebellum (Anterior Lobe), L LiG	838	0.001	0.126	5.07	−12	−58	−12
			0.228	4.80	−4	−64	−10
			0.362	4.57	12	−58	−22
**2. Association with severity of CAINS Alogia**							
L IFG triangularis, L MFG, L/R SMA, L PMd/PMv	4423	<0.001	0.001	7.00	6	38	48
			0.001	6.97	2	18	50
			0.003	6.60	−10	22	56
L/R ACC, pre-ACC	1229	<0.001	0.003	6.60	−2	32	2
			0.030	5.68	0	42	0
			0.160	4.97	4	40	12
L Cerebellum (Anterior Lobe), LiG	475	0.014	0.007	6.24	−10	−58	−10
			0.411	4.50	−32	−72	−10
			0.496	4.39	−6	−66	−8
L PMd/Pmv	499	0.012	0.012	6.05	−26	−24	62
			0.038	5.58	−16	−30	66
			0.863	3.92	−40	−34	52
R PMd/PMv	409	0.025	0.043	5.53	22	−26	66
			0.122	5.09	16	−16	70
			0.796	4.03	6	26	74
R Insula, Putamen, Pallidum, Caudate, NAcc	500	0.011	0.126	5.07	30	−16	−2
			0.573	4.30	24	18	−14
			0.659	4.20	34	8	−10
R Temporal pole, STG	352	0.042	0.187	4.90	58	14	−16
			0.547	4.33	52	6	−8
R Cerebellum (Posterior Lobe)	1344	<0.001	0.254	4.75	34	−70	−50
			0.277	4.71	24	−50	−48
			0.322	4.64	26	−74	−50
R PoG, STG, Ronlandic operculum	463	0.016	0.303	4.67	60	−28	18
			0.323	4.63	52	−16	8
			0.941	3.76	50	−10	−4
L SMG, angular gyrus	423	0.022	0.438	4.47	−58	−58	38
			0.559	4.32	−62	−50	30
			0.730	4.11	−54	−44	44
L Cerebellum (Posterior Lobe)	504	0.011	0.514	4.37	−40	−78	−46
			0.725	4.12	−46	−70	−46
			0.882	3.89	−30	−50	−46
**3. Association with severity of CAINS Blunted Affect**							
L/R Cerebellum (Anterior lobe), R FFG	507	0.013	0.191	4.87	12	−56	−22
			0.335	4.59	10	−36	−22
			0.517	4.35	0	−64	−12

Multiple regression within patients: Diminished Expression (1), Alogia (2), Blunted Affect (3).

*SMA* Supplementary motor area, *LiG* Lingual gyrus, *IFG* Inferior frontal gyrus, *MFG* Middle frontal gyrus, *PMd* Dorsal premotor area, *PMv* Ventral premotor area, *ACC* Anterior cingulate cortex, *NAcc* Nucleus Accumbens, *STG* Superior temporal gyrus, *PoG* Postcentral gyrus, *SMG* Supramarginal gyrus, *FFG* Fusiform gyrus. Covariates: age, six motion parameters, olanzapine equivalents, PANSS Positive subscore, duration of illness, years of education and diazepam equivalents.

## Discussion

Here we investigated the relationship between resting-state perfusion and negative symptom (NS) severity of the recently proposed NS factors and subdomains. As hypothesized, we observed increased perfusion in motor areas and the anterior lobe of the cerebellum to be associated with the factor diminished expression (DE). In addition, and in line with the hypothesis, we detected fronto-striatal resting-state perfusion associated with the subdomain alogia and perfusion of the right FFG to be associated with blunted affect. The results differ only marginally when applying either of the two NS scales SANS and CAINS. However, we found no association with brain perfusion and the second NS factor motivation and pleasure (MAP), or the subdomains anhedonia, asociality, and avolition. Our results suggest hyperactivation of motor areas and fronto-striatal brain areas to contribute to DE in patients with schizophrenia and point to the importance of focusing on NS factors and subdomains to identify brain alterations that match pathophysiological concepts of DE^2,6,34,39^.

### Perfusion of motor areas is associated with the NS factor DE of emotion and speech

Our main results show that patients with DE of emotion and speech present with higher perfusion within the supplementary motor area (SMA), the pre-SMA and the cerebellum. The SMA, and the most anterior portion commonly termed pre-SMA, are key players of the motor system and have repeatedly been shown to be associated with motor symptoms in schizophrenia^40–42^. In fact, there is considerable conceptual overlap of negative symptoms and motor symptoms in schizophrenia^43–46^. In particular, DE includes psychomotor symptoms of slowing such as decreased spontaneous movements and reduced facial expression. Not surprisingly an overlap in brain alterations relevant for negative symptoms and psychomotor symptoms has been suggested^47,48^. Hence our finding of increased SMA/pre-SMA perfusion at rest fits to the suggested key role of the SMA/pre-SMA in psychomotor symptoms in schizophrenia^41^ and the suggested role of motor symptoms for NS^43–46,49,50^.

Distinct structural and functional alterations in the motor system are associated with specific categories of motor dysfunction in schizophrenia^51^. In particular, the SMA is involved in selecting, preparing, and executing different modes of action. The SMA is via direct and indirect motor pathways tightly connected to the primary motor cortex, the pre-SMA, the striatum, the subthalamic nucleus (STN), the thalamus, and the corticospinal tract^52^. By contrast, the pre-SMA has extensive pre-frontal connectivity. Most importantly, SMA activity controls ongoing action via basal ganglia loops. The pre-SMA plays a critical role in exerting control over voluntary actions in situations of response conflict^52^. As a consequence, SMA and pre-SMA hyperperfusion may contribute to slowness in planning and execution of fine motor tasks and general hypokinesia^41,42,53–55^ and consequently NS such as decreased spontaneous movements and reduced facial expression^56,57^. Additionally, hyperperfusion of these key players of the motor system may contribute to higher motor impairments such as reduced gesturing as a further symptom of the NS factor DE. In detail, schizophrenia patients displayed hypoactivation during execution of familiar gestures within the premotor cortices (bilateral SMA, pre-SMA and cingulate motor areas)^58^. However, altered gesture production in schizophrenia has in addition been associated with altered activity of the praxis network^58–60^. Apart from the above mentioned key role in the motor system evidence suggests the pre-SMA/SMA to be highly relevant for cognitive functions such as attention, temporal processing^61–64^, problem solving and working memory capabilities in schizophrenia^65,66^, as well as in early psychosis^67,68^. Finally SMA dysfunction has been associated with altered sense of agency^52,69–71^.

The detected relatively large cluster within the cerebellum associated with DE includes most prominently the anterior lobe as well as the posterior lobe and the cerebellar vermis. In general, the role of the cerebellum in motor functions such as planning, coordination and fine tuning is well known and widely accepted^72^. In addition more recently, the relevance of cerebellar networks in emotional and cognitive processing such as timing and associative learning^73^, as well as, motivational and social behavior with regional specification has been discussed^74,75^. Both, the anterior (largely lobule V, but also lobules IV, and VI) and inferior posterior parts of the cerebellum (lobules VIIIa and VIIIb) are connected to the primary motor cortex and have been associated with sensorimotor processes, while the lateral and superior posterior cerebellum is suggested to be relevant for cognition and emotional processing^47,76–78^. In addition, the cerebellar vermis has also been linked to affective processing^76,78^. Bernard and Mittal advocate alterations within distinct motor and non-motor closed-loop circuits with the cerebral cortex as affected in schizophrenia^79^. Particularly, the shown alterations within the anterior and inferior posterior parts of the cerebellum may impact cerebellar-mediated motor behaviors. Thus, the cerebellar hyperperfusion may contribute to motor symptoms, and in addition reflect alterations in cerebellar networks relevant for emotional, cognitive and motivational behavior associated with DE. Likewise, some evidence also suggests involvement of the cerebellum in hallucinations and formal thought disorder^80^.

Finally, independent of NS factors altered brain activity of the SMA and the cerebellum has previously been suggested to be associated with overall negative symptoms. For instance, Vanes and colleagues showed reduced cerebellar and SMA task-based fMRI activation during a Stroop task associated with negative symptoms severity in particular in patients suffering from a first schizophrenia episode^67^. Independent of NS factors, Bernard and colleagues advocate that cerebellar motor networks might contribute to overall negative symptoms in schizophrenia patients^81^.

Taken together the detected hyperperfusion of the SMA and the cerebellum nicely fit to the framework on motor symptoms in schizophrenia^41^ and findings of studies investigating motor abnormalities and gesturing in schizophrenia^42,45,58–60^ as a highly relevant aspect of the NS factor DE^5,8,28,44^.

Importantly these findings fit to evidence showing an association of NS, motivation and motor symptoms at the behavior level in schizophrenia. In fact, patients with more negative symptoms showed reduced physical activity^45,82^, physical activity is reduced during the course of the disorder^83^, autonomous motivation was associated with physical^84^, and physical activity predicted the course of overall negative symptoms between episodes^85^.

### Perfusion of motor areas, fronto-striatal areas and the FFG associated with the NS subdomains blunted affect and alogia

When looking at the association of brain perfusion and NS subdomains we detected effects associated with the subdomains suggested to build the DE factor (blunted affect and alogia). Not surprisingly, our results therefore mirror the described effects detected with DE. In particular, showing hyperperfusion in the SMA, the ventral premotor (PMv) and the dorsal premotor area (PMd) associated with alogia and hyperperfusion within the cerebellum associated with alogia and blunted affect. Moreover, additional brain areas emerged. Specifically, severe blunted affect was besides associated with perfusion of the right FFG. Severe symptoms of alogia were in addition associated with fronto- and paralimbic resting-state perfusion.

The role of the SMA and the cerebellum for NS has been discussed above. Likewise, the few studies on NS subdomains suggest the motor cortex to be associated with blunted affect. Lee and colleagues showed for instance severity of blunted affect to be associated with aberrant brain activity in premotor and motor cortex and the inferior parietal lobule using a facial emotion imitation task^15^. In line with previous imaging reports assessing task-based activity, severe symptoms of blunted affect were associated with increased perfusion at rest of the right FFG^12–14^. Although the exact functionality of the FFG is still disputed, there is relative consensus on its involvement in face emotion processing^86,87^, on which schizophrenia patients show deficits^86,87^ and NS are presumed to have influence^88^. In fact, facial recognition and memory difficulties were associated with severity of overall NS^89,90^, reduced FFG volume correlated significantly with higher NS but not with positive symptoms^90,91^, and patients with persistent NS show smaller GM volume of the right FFG compared to patients with non-persistent NS^92^. Consistent with these results evidence from PET (lower glucose metabolic rate in the FFG) and EEG (event related potential components of the left FFG cluster at P100) suggest overall NS to be associated with FFG activity^88,93^. However, other reports focusing on hallucinations advocate the relevance of the FFG for pathophysiological concepts on hallucinations^94^. Thus, our results fit to the suggested substantial role of the FFG in the development of NS^91^ and further specify the relevance for the NS subdomain blunted affect. In fact, it seems plausible that in particular reduced eye contact, one aspect of the NS subdomain blunted affect, may lead to deficits in facial recognition. The detected hyperactivation of the FFG may reflect a compensatory effect. However, this remains speculative, as it was not directly tested in the present study.

As stated and consistent with the NS factor DE alogia, the second subdomain of DE, was associated with hyperperfusion of the SMA and the cerebellum. In addition, alogia was linked to increased perfusion in the frontal cortex (IFG, MFG, ACC), motor and premotor areas (SMA, pre-SMA, the dorsal and ventral premotor area), basal ganglia and limbic and paralimbic brain areas including the insula, putamen, pallidum, caudate and nucleus accumbens (Table 1, Fig. 2). To our knowledge, so far no study tested whole brain perfusion associated with NS subdomains. In line with our results areas relevant for cognitive and emotional processing in motor and fronto-limbic networks as well as the caudate nuclei have been suggested to play an essential role in the pathophysiology of the NS factor DE^12–19,28^. When particularly looking at the subdomain alogia first task-based MRI evidence suggested alogia to be linked to abnormal activity within the anterior cingulate cortex (ACC)^17^ and areas of the basal ganglia, including pallidum and caudate^18^. In detail, Hager and colleagues investigated the neural effects of monetary reward performance using an n-back fMRI task. Their results revealed a negative correlation between severity of DE using the SANS NS scale and brain activity in the rostral ACC. Considering the role of the ACC in cognitive functioning including emotional processing, attention allocation and mood regulation, the authors advocate that especially symptoms in DE lead to difficulties in cognitive processing. This and our results support the cognitive resource limitation model proposing that emotional expression requires a considerate amount of cognitive resources^95^. These resources are thought to be insufficient for emotional expression in patients particularly during high cognitive demanding situations such as social interactions. Thus we provide additional evidence for the cognitive resource limitation model relevant for DE in schizophrenia supporting studies connecting cognitive impairment and DE^9^. Although prior evidence linking activation in the insula or the STG to the NS subdomain alogia is limited the detected link fits to previous reports showing functional and structural alterations in the insula cortex, the STG and overall negative symptom severity^90,96–103^. In addition, early evidence shows that insular lesions can lead to deficits in speech initiation and planning as well as emotional expression and comprehension^104–106^. Finally, the key role of the STG in language processing is widely accepted^107^. Therefore, an association with the NS subdomains involved in speech initiation (alogia) is plausible^102^.

Interestingly examining perfusion associations of the two consensus subdomains of DE (blunted affect and alogia) separately revealed distinct neural correlates between the two subdomains. This points to the fact that indeed and as recently suggested looking at subdomains may help identifying specific underlying mechanism^34–36^.

### No association with the NS factor MAP and the subdomains anhedonia, asociality or avolition

Contrary to previous reports, we did not find any significant associations between perfusion and MAP severity including its consensus subdomains anhedonia, asociality or avolition. Brain abnormalities in corticostriatal areas have been linked to reward anticipation and motivation and its subdomains anhedonia, asociality and avolition^6^. We thus hypothesized an association between brain perfusion in prefrontal-striatal areas and severity of MAP, as suggested by numerous studies^20–23,26^ and meta-analysis^24^. One reason why the results do not support our assumption might be relevant methodological differences compared to previous reports. In particular, the majority of the previous studies examined differences in the blood-oxygen-level-dependent (BOLD) signal between rewarding and non-rewarding stimuli while we looked at whole brain absolute activations at rest using ASL. In fact only one previous study focused on investigating the association of NS factors and rCBF and observed a significant positive correlation between MAP and perfusion in the striatum^38^. In contrast to our study, Schneider and colleagues, however, used a region-of-interest approach focusing on the striatum.

### Limitations

Our results extend previous findings detecting resting-state hyperperfusion in motor and fronto-limbic areas linked to DE in schizophrenia considering recent guidelines on the assessment of negative symptoms. Particularly, we used whole brain ASL to reflect absolute brain metabolism. However, some limitations require discussion. First, we cannot rule out possible effects of medication on our findings of altered rCBF^108^. In our sample, all but four patients were treated with antipsychotic medication at the time of scanning. In fact, reports on the impact of antipsychotic medication on rCBF yielded conflicting results. While some studies detect no effects of medication on rCBF^109,110^, others show CBF reduction^111^, regional different effects on rCBF^112^ or substance depending effects on rCBF^113^. However, we solely included patients on stable medication treated with first- or second-generation antipsychotics. In addition, we included antipsychotic medication dosage (OLZs) and dosage of benzodiazepines (diazepam equivalents), as a covariate of no-interest in all our analyses. Furthermore, as expected factors of NS were partially overlapping. This hampers the sharp distinction of effects of NS factors. Additionally, we used two independent measures resulting in marginal differences of the results. These differences may result from limitations of the first-generation measurement SANS. However we directly addressed these weaknesses by applying the most recent conceptualization of NS^34–36^ and implemented an improved rating of the SANS to measure NS factors. Finally, we cannot comment on possible associations of the detected brain activity (i.e. within the pre-SMA / SMA) with cognitive functions or the performance of neurocognitive tasks as this was not specifically tested in the study.

To conclude, investigating the association of NS factors or subdomains and resting-state whole brain perfusion revealed effects for DE and the subdomains blunted affect and alogia. In particular, we highlight the central role of motor areas for DE in schizophrenia. Furthermore, our results match the suggested relevance of fronto-limbic networks and the FFG in the development of NS and further specify the FFG to be key for the NS subdomain blunted affect. Our results support the relevance of the recently proposed NS factors and subdomains to unravel brain perfusion alterations that match pathophysiological concepts of NS. Future multimodal studies may further disentangle the link of task and resting-state based brain alterations associated with NS factors and subdomains in schizophrenia^114–116^. Finally, in-depth studies on NS pathophysiology will pave the way for novel treatment strategies, such as non-invasive brain stimulation techniques.

## Methods

### Participants

We included 47 patients with schizophrenia spectrum disorders and 44 age-and sex matched healthy controls. We diagnosed patients according to the DSM-5 diagnostic and statistical manual of mental disorders. Demographic and clinical characteristics are given in Table 2.

**Table 2:** Demographic and clinical variables.

	HC (*n* = 44)	SZ (*n* = 47)	df	T/X^2^	*P*
Sex (% male)	62%	59%	1	0.065	0.799
Age (years)	38.8 ± 13.58	38.2 ± 11.38	89	−0.230	0.819
Education (years)	14.1 ± 2.66	13.4 ± 3.09	89	−1.190	0.237
DOI (years)	–	12.2 ± 12.25	–		
Number of episodes	–	6.6 ± 7.06	–		
OLZ	–	10.29 ± 8.85	–		
PANSS total	–	72.6 ± 17.12	–		
PANSS pos	–	18.2 ± 6.43	–		
PANSS neg	–	18.4 ± 5.06	–		
CAINS DE	–	0.8 ± 0.86	–		
CAINS MAP	–	1.7 ± 0.90	–		
CAINS Blunted Affect	–	1.0 ± 0.97	–		
CAINS Alogia	–	0.6 ± 0.97	–		
CAINS Anhedonia	–	1.7 ± 0.98	–		
CAINS Avolition	–	1.8 ± 1.05	–		
CAINS Asociality	–	1.5 ± 0.88	–		

Values presented as mean ± standard deviation (SD). *DOI* Duration of illness; *OLZ* Olanzapine equivalents, *PANSS* Positive and Negative Syndrome Scale, *pos* positive, *neg* negative, *CAINS* Clinical Assessment Interview for Negative Symptoms, *DE* Diminished Expression, *MAP* Motivation and Pleasure, *HC* Healthy Controls, *SZ* Schizophrenia Patients.

Schizophrenia patients were recruited from the inpatient and outpatient department of the University Hospital of Psychiatry and Psychotherapy in Bern, Switzerland. Exclusion criteria for all participants included head trauma or general exclusion criteria for MRI scans (e.g. metallic implants, claustrophobia and pregnancy), and a history of substance abuse or dependence other than nicotine as assessed during the patients’ interviews, careful chart review, and according the mini international neuropsychiatric interview (MINI)^117^. Additionally, exclusion criteria for healthy controls were history of any psychiatric disorder or any first-degree relatives with schizophrenia spectrum disorders according to the MINI^117^. We obtained written informed consent from all participants. The study protocol adhered to the declaration of Helsinki and was approved by the local Ethics Committee, Bern (KEK). All participants included in this study participated in previous studies^58,118^. We assessed antipsychotic medication and calculated olanzapine equivalents (OLZs) in accordance to Leucht et al^119^. All but four patients were treated with antipsychotic medication at the time of testing. Diazepam equivalents were calculated according to Ashton^120^.

### Clinical assessments

We assessed symptom severity in patients using the Positive and Negative Syndrome Scale (PANSS)^121^, the Clinical Assessment Interview for Negative Symptoms (CAINS)^57^ and the Scale for the Assessment of Negative Symptoms (SANS)^56^.

The CAINS interview was particularly designed to assess the five negative symptom consensus subdomains (blunted affect, alogia, anhedonia, asociality and avolition). All 13 items are scored on a five-point scale ranging from 0 (no impairment) to 4 (severe deficit). We used the expert ratings of each single item of the CAINS to calculate means for the five subdomains according to Strauss and colleagues^34^. Next, we used the mean of alogia and blunted affect to calculate the mean of diminished expression (DE) and the mean of anhedonia, asociality and avolition to calculate the mean of motivation and pleasure (MAP) as these form the two main suggested negative symptom factors (Table 2).

Likewise, the SANS is an interview-based rating scale for negative symptoms. It consists of five subscales: affective flattening or blunting, alogia, avolition-apathy, anhedonia-asociality, and attention. Within each subscale, experts rate severity of 3–8 single symptoms from 0 (none) to 5 (severe). In accordance with the most recent guidelines, we removed SANS items with redundant or conflicting content to the current concept of NS (inappropriate affect, poverty of content of speech and inattention). Next, we calculated mean values for each of the five consensus subdomains^34,36^ and again calculated means of the two main negative symptom factors DE and MAP. In particular, the DE factor was calculated using the mean of blunted affect and alogia. MAP was calculated using the mean of anhedonia, asociality and avolition.

We provide means and standard deviations of the NS factors (DE and MAP) and the consensus subdomains (blunted affect, alogia, anhedonia, asociality and avolition) for the CAINS in Table 2 and ranges depicting negative symptom severity for both rating scales in the supplement (Table S3).

### MRI acquisition

We performed structural and functional MRI on a 3 T MRI scanner (Siemens Magnetom Trio; Siemens Medical Solutions, Erlangen, Germany) using a 12-channel radio frequency head coil for signal reception. For all participants we obtained structural 3D-T1-weighted (Modified Driven Equilibrium Fourier Transform Pulse Sequence; MDEFT) images: 176 sagittal slices with 256 × 256 matrix points with a non-cubic field of view (FOV) of 256 mm, yielding a nominal isotopic resolution of 1 mm³ (i.e. 1 mm × 1 mm × 1 mm). Scanning parameters further contained, repetition time (TR) of 7.92 ms, echo time (TE) of 2.48 ms and a flip angle (FA) of 16°.

In order to obtain 110 functional images per participant, we applied a pseudo continuous arterial spin labeling (pCASL) sequence. This sequence provided 20 slices in ascending order, 64 × 64 matrix points with a non-cubic 230 mm FOV, yielding a nominal isotopic resolution of 4.27 mm³ (i.e. 3.6 mm × 3.6 mm × 6 mm), TR of 4000 ms, TE of 18 ms and a FA of 25°^122^.

### Data processing

We analyzed structural and perfusion imaging data using SPM version 12 (The Wellcome Centre for Human Neuroimaging, UCL Queen Square Institute of Neurology, London, UK; https://www.fil.ion.ucl.ac.uk/spm/). We applied an in-house written MATLAB program toolbox to preprocess perfusion images^42,118,122,123^. Briefly, we realigned the ASL images and implemented a voxel-wise calculation of the mean regional resting-state cerebral blood flow (rCBF). We stored these rCBF maps for each participant. Further, we applied coregistration of all rCBF maps to the structural images. Finally, we normalized and smoothed the data with 8 mm full-width at half-maximum (FWHM) kernel.

### Statistical analyses

We analyzed demographic and clinical data using SPSS version 28 (SPSS Inc., Chicago, IL, USA). To test group differences in demographic and clinical variables we used two-sample *t-*tests and chi-square tests (*χ*^*2*^).

Our main analysis focused on the effect of NS factors on rCBF. Therefore, we applied two separate multiple regression analyses in SPM to test the association between brain perfusion and mean severity of DE and MAP as calculated from the CAINS. To test for consistency of the results we repeated the analyses for the second negative symptom scale (SANS), again within two separate multiple regression analyses.

In addition to the two main factors, we explored the association of NS severity within the five consensus subdomains. Again, we calculated the association between mean severity for each subdomain and rCBF by applying multiple regression analyses for both negative symptom scales. We thus performed exploratory analyses with five separate multiple regression analyses for the CAINS as well as for the SANS. Finally, to test group differences in rCBF independent of NS we performed a *t*-test comparing patients and controls.

For all imaging analyses, we included age, six motion parameters, olanzapine equivalents, PANSS positive subscore, duration of illness, years of education and diazepam equivalents as covariates of no-interest. In addition, voxels with <10 [ml/100 g/min] blood flow were excluded from all analyses to exclude voxels in white matter^118^. We applied a statistical threshold of *p* < 0.05 family wise error corrected for multiple testing (FWE-corr). We present results for the Clinical Assessment Interview for Negative Symptoms (CAINS) corrected at peak- or cluster level in the main manuscript. Additionally, results for the Scale for the Assessment of Negative Symptoms (SANS) corrected for multiple testing (FWE-corr) at peak- or cluster level are presented in the supplementary material (Table S1). We produced figures using SPM12 and the SPM xjView toolbox (https://www.alivelearn.net/xjview). For illustration purposes, we produced images at *p* < 0.001 uncorrected with a minimum cluster size of 350 voxels. The minimum cluster size of 350 voxels was chosen to guarantee that only brain regions surviving the strict FWE corrections <0.05 are displayed. We extracted the mean perfusion values post-hoc from significant clusters for all patients and healthy controls using MarsBaR^124^ and plotted extracted brain perfusion values and NS severity.

### Supplementary information

Supplementary Material

## Data Availability

The statistical maps that support the findings of this study are available from the corresponding author upon reasonable request. The data are not publicly available due to them containing information that could compromise research participant privacy or consent. Explicit consent to deposit raw-imaging data was not obtained from the patients.
