# Supplementary material for: Resting-state perfusion in motor and fronto-limbic areas is linked to diminished expression of emotion and speech in schizophrenia
Source: Schizophrenia (Heidelb). 2023 Aug 12;9(1):51. doi: 10.1038/s41537-023-00384-7 (PMC10423240; doi:10.1038/s41537-023-00384-7)
Supplement: Supplementary file 1 — Supplementary Material [file 41537_2023_384_MOESM1_ESM.docx]

**Supplementary material:**

**Resting-state perfusion in motor and fronto-limbic areas is linked to diminished expression of emotion and speech in schizophrenia**

Nicole Gangl^a,*^, MSc, Frauke Conring^a^, MSc, Andrea Federspiel^a^, PhD, Roland Wiest^b^, MD, Sebastian Walther^a*^, MD, Katharina Stegmayer^a*^, MD

^a^Translational Research Center, University Hospital of Psychiatry and Psychotherapy, Bern, Switzerland

^b^Support Center of Advanced Neuroimaging (SCAN), University Institute of Diagnostic and Interventional Neuroradiology, Inselspital, Bern, Switzerland

***Sebastian Walther and Katharina Stegmayer contributed to this work equally**

*To whom correspondence should be addressed: Nicole Gangl, University Hospital of Psychiatry, Bolligenstrasse 111, 3060 Bern, Switzerland; Tel: +41-31-930-8717, Fax: +41-31-930-9404, e-mail: [nicole.gangl@upd.unibe.ch](mailto:nicole.gangl@upd.unibe.ch)

| **Table S1.** Perfusion in brain regions associated with dimensions and consensus domains of negative symptoms according to SANS. Multiple regression within patients: Diminished Expression (1), Alogia (2). | | | | | | | |
| --- | --- | --- | --- | --- | --- | --- | --- |
| 1. **Association with severity of SANS Diminished Expression** | | | | | | | |
| Brain region |  | *P* (FWE-corr) | |  | | | |
|  | k_E_ | Cluster | Peak | T | MNI coordinates  (x, y, z) | | |
| No significant clusters | | | | | | | |
| 1. **Association with severity of SANS Alogia** | | | | | | | |
| Brain region |  | *P* (FWE-corr) | |  | | | |
|  | k_E_ | Cluster | Peak | T | MNI coordinates  (x, y, z) | | |
| L/R MFG, L PoG, L PMd/PMv, L/R SMA, L ACC | 3928 | <0.001 | 0.014 | 6.02 | -2 | 16 | 46 |
|  |  |  | 0.022 | 5.83 | -52 | -16 | 44 |
|  |  |  | 0.025 | 5.77 | -22 | -18 | 66 |
| L MOG, cuneus | 458 | 0.013 | 0.028 | 5.72 | -20 | -92 | 6 |
|  |  |  | 0.643 | 4.24 | -42 | -74 | 2 |
|  |  |  | 0.878 | 3.92 | -10 | -96 | 10 |
| R STG, PoG | 400 | 0.023 | 0.086 | 5.26 | 56 | -32 | 6 |
|  |  |  | 0.660 | 4.22 | 62 | -16 | 28 |
|  |  |  | 0.901 | 3.88 | 62 | -30 | 16 |
| L/R Cerebellum (Anterior Lobe), L LiG, L posterior cingulate, L precuneus | 2536 | <0.001 | 0.154 | 5.01 | 12 | -58 | -24 |
|  |  |  | 0.221 | 4.84 | -10 | -46 | 32 |
|  |  |  | 0.335 | 4.64 | -16 | -60 | -4 |
| R SMG, angular gyrus | 385 | 0.026 | 0.575 | 4.32 | 46 | -42 | 30 |
|  |  |  | 0.785 | 4.06 | 60 | -48 | 28 |
|  |  |  | 0.912 | 3.85 | 56 | -58 | 34 |
| MFG = Middle frontal gyrus; PoG = Postcentral gyrus; PMd = Dorsal premotor area; PMv = Ventral premotor area; SMA = Supplementary motor area; ACC = Anterior cingulate cortex; MOG = Middle occipital gyrus; STG = Superior temporal gyrus; LiG = Lingual gyrus; SMG = Supramarginal gyrus. Covariates: Age, Six motion parameters, Olanzapine equivalents, PANSS Positive subscore, Duration of illness, Years of education and diazepam equivalents. | | | | | | | |

| **Table S2.** Differences between patients with schizophrenia and healthy controls in whole brain resting-state-state perfusion. | | | | | | | |
| --- | --- | --- | --- | --- | --- | --- | --- |
| ***t*-test healthy controls > patients with schizophrenia:** | | | | | | | |
| Brain region |  | *P* (FWE-corr) | |  |  | | |
|  | k_E_ | Cluster | Peak | T | MNI coordinates  (x, y, z) | | |
| L planum temporale, parietal operculum, STG | 7 | 0.022 | 0.024 | 5.08 | -58 | -34 | 14 |
| STG =Superior temporal gyrus. Covariates: Age, Six motion parameters, Years of education. Threshold was set to *p* < 0.05 FWE-corrected. | | | | | | | |

| **Table S3.** Negative Symptom Severity in patients with schizophrenia according to the Clinical Assessment Interview of Negative Symptoms (CAINS) and the Scale for the Assessment of Negative Symptoms (SANS). | | | | | | | | | |
| --- | --- | --- | --- | --- | --- | --- | --- | --- | --- |
|  |  |  |  |  |  |  |  |  |  |
| **Negative Symtom Severity** | |  | **Diminished Expression** | **Motivaton and Pleasure** | **Blunted Affect** | **Alogia** | **Avolition** | **Anhedonia** | **Asociality** |
|  |  |  |  |  |  |  |  |  |  |
|  |  | range | N | N | N | N | N | N | N |
| **CAINS** | **0 = No impairment** | .00-0.49 | 21 | 6 | 20 | 32 | 4 | 7 | 3 |
|  | **1 = Mild deficit** | 0.50-1.49 | 13 | 13 | 14 | 7 | 9 | 14 | 20 |
|  | **2 = Moderate deficit** | 1.50-2.49 | 10 | 17 | 8 | 4 | 18 | 14 | 14 |
|  | **3 = Moderately severe deficit** | 2.50-3.49 | 3 | 11 | 5 | 4 | 11 | 12 | 10 |
|  | **4 = Severe deficit** | 3.50-4.00 | 0 | 0 | 0 | 0 | 5 | 0 | 0 |
| **SANS** | **0 = None** | .00-0.49 | 22 | 6 | 14 | 32 | 9 | 8 | 7 |
|  | **1 = Questionable** | 0.50-1.49 | 13 | 14 | 12 | 10 | 11 | 2 | 19 |
|  | **2 = Mild** | 1.50-2.49 | 7 | 11 | 12 | 1 | 13 | 17 | 11 |
|  | **3 = Moderate** | 2.50-3.49 | 3 | 15 | 6 | 3 | 9 | 13 | 8 |
|  | **4 = Marked** | 3.50-4.49 | 2 | 1 | 3 | 1 | 5 | 7 | 2 |
|  | **5 = Severe** | 4.50-5.00 | 0 | 0 | 0 | 0 | 0 | 0 | 0 |
